# Improved industrial induction time-based technique for evaluating kinetic hydrate inhibitors

**DOI:** 10.3389/fchem.2024.1396862

**Published:** 2024-05-22

**Authors:** Mahboobeh Mohammad-Taheri, Bahman Tohidi, Bahram Ghanbari, Zahra Taheri Rizi

**Affiliations:** ^1^ Chemical Polymeric and Petrochemical Technology Development Research Division, Research Institute of Petroleum Industry, Tehran, Iran; ^2^ Institute of GeoEnergy Engineering, Heriot-Watt University, Edinburgh, United Kingdom; ^3^ Presently Guest Research at the Copernicus, Universiteit Utrecht, Utrecht, Netherlands; ^4^ Hydrafact Ltd., Edinburgh, United Kingdom; ^5^ Department of Chemistry, Sharif University of Technology, Tehran, Iran

**Keywords:** kinetic hydrate inhibitor, induction time-based method, hydrate equilibrium point, inhibitory performance, hydrate memory effect method, water-hydrate-memory, IT method, HME method

## Abstract

Kinetic hydrate inhibitor laboratory testing before field application is one of the key priorities in the oil and gas industry. The common induction-time-based technique is often used to evaluate and screen for kinetic hydrate inhibitors (KHIs). However, the main challenge relates to the stochastic nature of hydrate nucleation observed in fresh systems, which often results in scattered data on hydrate formation with unacceptable uncertainties. A much more precise KHI evaluation method, called crystal growth inhibition (CGI), provides comprehensive insights into the inhibitory behavior of a kinetic hydrate inhibitor, including both hydrate formation and decomposition. Given that industry does not require this much information, it is not feasible to expend either much time or cash on this strategy. This study aims to provide a cost-effective technique that presents maximum data accuracy and precision with relatively little time and cost expenditure. Hence, the impact of water-hydrate memory on improving the accuracy and repeatability of the results of the induction-time-based technique (IT method) was examined. First, the concept of water-hydrate memory, which contains information about how it is created, was reviewed, and then, the factors influencing it were identified and experimentally investigated, like the heating rate of hydrate dissociation and the water-hydrate memory target temperature during heating. Finally, a procedure was developed based on the background information in the earlier sections to compare the consistency of the results, originating from the conjunction of water-hydrate memory with the IT technique. The results of replications at KHI evaluation target temperatures of 12.3–12.4°C and 11.5–11.7°C showed that more repeatable data were obtained by applying water-hydrate memory, and a more conclusive decision was made in evaluating KHI performance than with an IT method. It seems that combining the IT method with water-hydrate memory, introduced as the “HME method”, can lead to more definitive evaluations of KHIs. This approach is expected to gain in popularity, even surpassing the accurate but complex and time-consuming CGI method.

## 1 Introduction

Hydrate inhibitors are commonly used in the gas and oil industry to mitigate the risk of hydrate formation. Since it is crucial to use a strategy that minimizes use of the incorrect inhibitor as precisely and quickly as possible, several studies have been conducted to propose techniques for assessing the effectiveness of hydrate inhibitors. However, the accurate simulation of fluid conditions in the laboratory poses several challenges. For example, once a hydrate forms in a transportation pipeline, the next flow-assurance activities attempt to remove the hydrate blockage, resulting in the water-hydrate memory being left in the aqueous phase (Park et al., 2013). The “water-hydrate-memory” phenomenon and its effect on hydrate formation was identified based on the history of experiments that used the Total company`s Hydrate Loop Apparatus. It was observed that if hydrate forms in a system for the first time and then completely decomposes due to an increase in temperature, while the temperature of the system does not rise much above the equilibrium temperature, the re-formation of the hydrate by re-cooling the system is much easier and occurs much faster (Peytavy et al., 2008). Then, unlike those without a history of hydrate formation, the risk of hydrate formation will increase in the pipeline, and screening techniques for kinetic hydrate inhibitors should also take this into account (Park et al., 2013).

However, the impact of the water-hydrate-memory phenomenon might also have to be considered with gas production from hydrate-bearing reservoirs. The gas temperature extracted from the decomposition of hydrate layers in the well is thus decreased by heat exchange with the environment after reaching the surface. This may increase the risk of hydrate re-formation, especially through offshore pipelines, and highlights the need to use a proper hydrate inhibitor (Kou et al., 2022).

Induction-time-based approaches are frequently employed to test the effectiveness of kinetic hydrate inhibitors. These strategies prolong the period required for the system to enter the hydrate area and for hydrate crystals to form—the induction time of hydrate formation. The primary drawback of this technique is the variability in the results, requiring multiple tests to provide a conclusive assessment of the efficacy of the kinetic hydrate inhibitor. Of course, the data uncertainty comes from the random and stochastic nature of primary hydrate nuclei formation.

According to our relative knowledge of the water-hydrate-memory phenomenon on hydrate nucleation, especially the effect of obtaining more reproducible data, Duchateau et al. presented a methodology based on implementing the water-hydrate-memory effect in laboratory autoclaves, with financial support from Total and Arkema/Ceca (Duchateau et al., 2008; Duchateau et al., 2009), and then used this method to investigate the possibility of ranking KHIs (Duchateau et al., 2010).

Tohidi and colleagues were also developing the crystal growth inhibition (CGI) method to investigate KHI performance directly on hydrate crystal growth based on use of some residue of hydrate crystals as hydrate precursors (Glénat et al., 2011; Ross et al., 2011; Tohidi et al., 2012). In this method, the hydrate growth pattern is drawn for each kinetic hydrate inhibitor (or missed with solvents) at a specific concentration (Mozaffar et al., 2016a; Mozaffar et al., 2016b). Although this method is very accurate in analyzing the behavior of KHIs *versus* hydrate growth, its time-consuming and complex nature should be taken into account for use in industrial applications.

Recently, the water-hydrate-memory phenomenon has attracted the attention of researchers. May et al. (2014) also tried to quantify the water-hydrate memory with the help of subcooling comparisons obtained by HP- ALTA (high-pressure automated lag time apparatus) experiments under quiescent conditions for different KHIs. An attempt was made to describe the water-hydrate-memory impact on hydrate formation using a computational kinetic model. This considered hydrate formation as an autocatalytic kinetic reaction, including nucleation and early growth. It found that the facilitation of the nucleation stage was indicated by a reduced induction time (Ke et al., 2020). However, it was reported that water-hydrate memory enhanced the number of moles of gas by hydrate formation compared to fresh water (Pallipurath et al., 2019). Moreover, the mechanisms of water-hydrate memory were investigated by experimentally determining the nucleation curves of hydrate formation (Wei and Maeda, 2023).

Despite this, it appears that water-hydrate memory can be employed most practically employed in conjunction with kinetic hydrate inhibitor evaluation methods in the gas and oil industries. Although the simple induction-time-based method costs more money and time, it was used widely in the evaluation of KHI performance before field tests. In this work, we attempted to introduce an improved industrial procedure for KHI evaluation to apply in a gas production field using the water-hydrate-memory phenomenon. The goal was to make the results more reproducible, removing the need for more test replication to investigate hydrate kinetic inhibitor performance.

## 2 Experiment

### 2.1 Material

The real sour gas (normalized composition given in Table 1) and liquid gas condensate used in the tests were sampled from a field pipeline. A commercial kinetic inhibitor (“KHI”) and a commercial corrosion inhibitor (“CI”) were used in the tests. Distilled water was used in preparing solutions.

**TABLE 1:** Normalized real sour gas composition.

Component	Mol%
N_2_	3.83
CO_2_	2.35
H_2_S	0.09
CH_4_	86.47
C_2_H_6_	4.91
C_3_H_8_	1.58
i-C_4_H_10_	0.30
n-C_4_H_10_	0.46
Total	100.00

### 2.2 Apparatus

**FIGURE 1:**
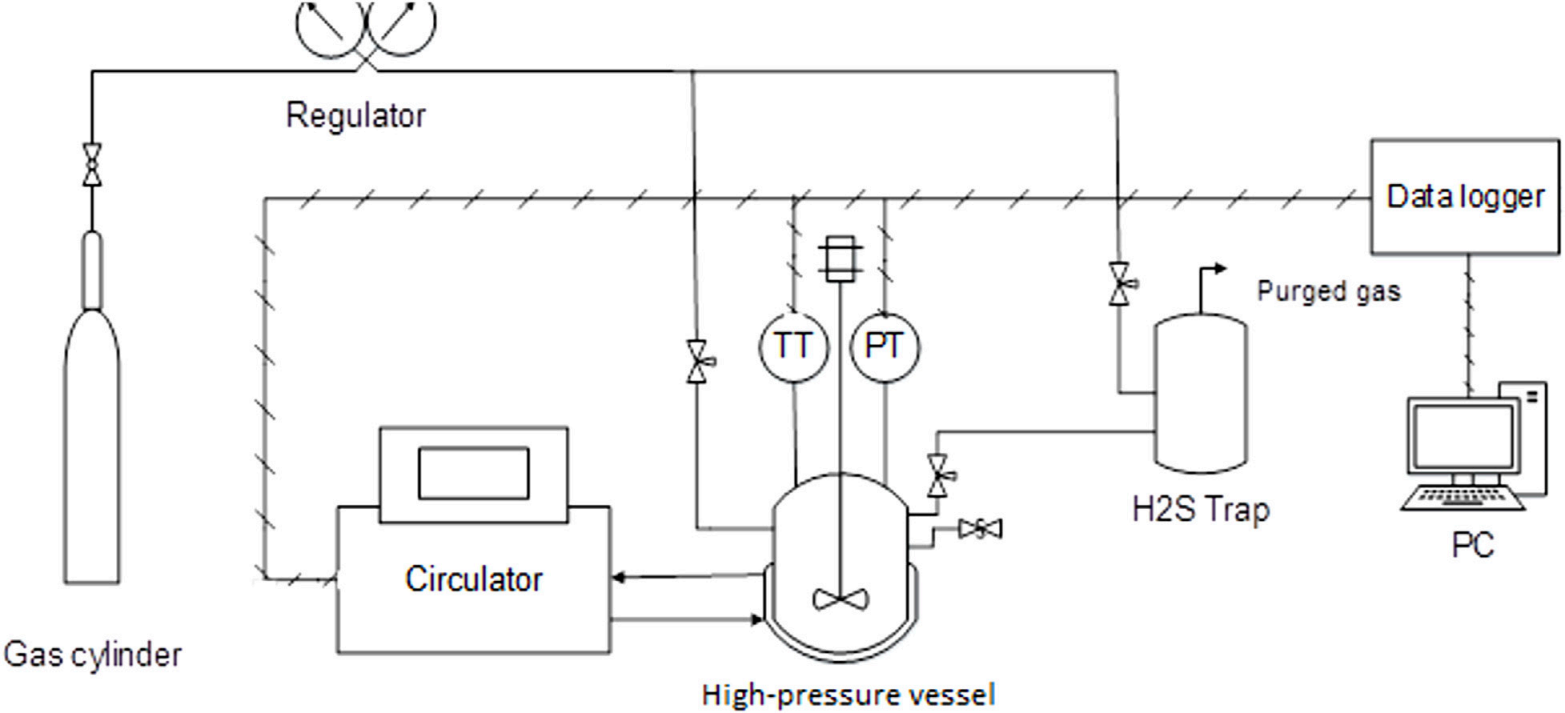
Scheme of the apparatus. (PT, pressure transducer; TT, temperature transducer).

Figure 1 illustrates the scheme of the experimental apparatus. A 750-mL stainless steel high-pressure vessel equipped with a magnetic-drive stirrer and thermal jacket was used. A programmable bath (circulator) adjusted the vessel temperature based on the test temperature program. A resistance temperature thermometer (PT-100) with an accuracy of 0.2°C and a digital pressure transducer with a 3.0 psi accuracy measured the vessel’s temperature and pressure during the test. A data logger recorded the data on a PC. An H_2_S trap was installed to safely release the gas into the atmosphere at the end of the test.

### 2.3 Method

#### 2.3.1 Commissioning section

Taken from a database of real subsea pipeline operational conditions, the liquid-to-gas and the water-to-hydrocarbon volumetric ratios were 40:60 and 1:6, respectively. The CI and KHI were added by 0.5 and 1.25 mass percent, respectively, of the water amount. Distilled water was used to prepare the aqueous phase. After cleaning, the 750-mL vessel was loaded with aqueous and field hydrocarbon as above and then pressurized up to 1,856.5 psi, with the field gas at 30°C, a condition in which gas and the two liquid phases were in thermodynamic stability with each other. Then, under isochoric condition, the system reached 1,668 psi pressure and 12°C.

#### 2.3.2 Hydrate equilibrium point determination

After the commissioning procedure in the absence of KHI, the vessel was quickly cooled due to the bath temperature reducing from 20°C to 5°C at a rate of 5°C/h. Once the hydrate formed quantitatively, stepwise heating of the system was followed to obtain the total decomposition of the hydrate in an equilibrium manner. Thus, a one-step increase in temperature, such as 0.5°C, was accompanied by an adequate amount of time for the system to stabilize its temperature and pressure; after this, a one-step increase in temperature was permitted. The heating and cooling curves converged with the decomposition of the final hydrate particle, and the corresponding temperature was termed the “hydrate equilibrium point”.

In this work, heating was done at two rates—fast and slow—to both conserve time and proceed the hydrate decomposition maintaining equilibrium. For a fast heating rate, the system was given 2 h for every 1°C to obtain stabilized temperature and pressure. In contrast, the system was given 4 h for every 0.5°C increase in bath temperature during the fast heating.

#### 2.3.3 Evaluation of hydrate risk in the presence of KHI

During all experiments, the creation of water-hydrate memory occurred initially through the hydrate’s formation from the fresh liquid system, followed by the decomposition of the hydrates at a temperature above the equilibrium point.

## 3 Results and discussion

### 3.1 Determining the hydrate equilibrium point

**FIGURE 2:**
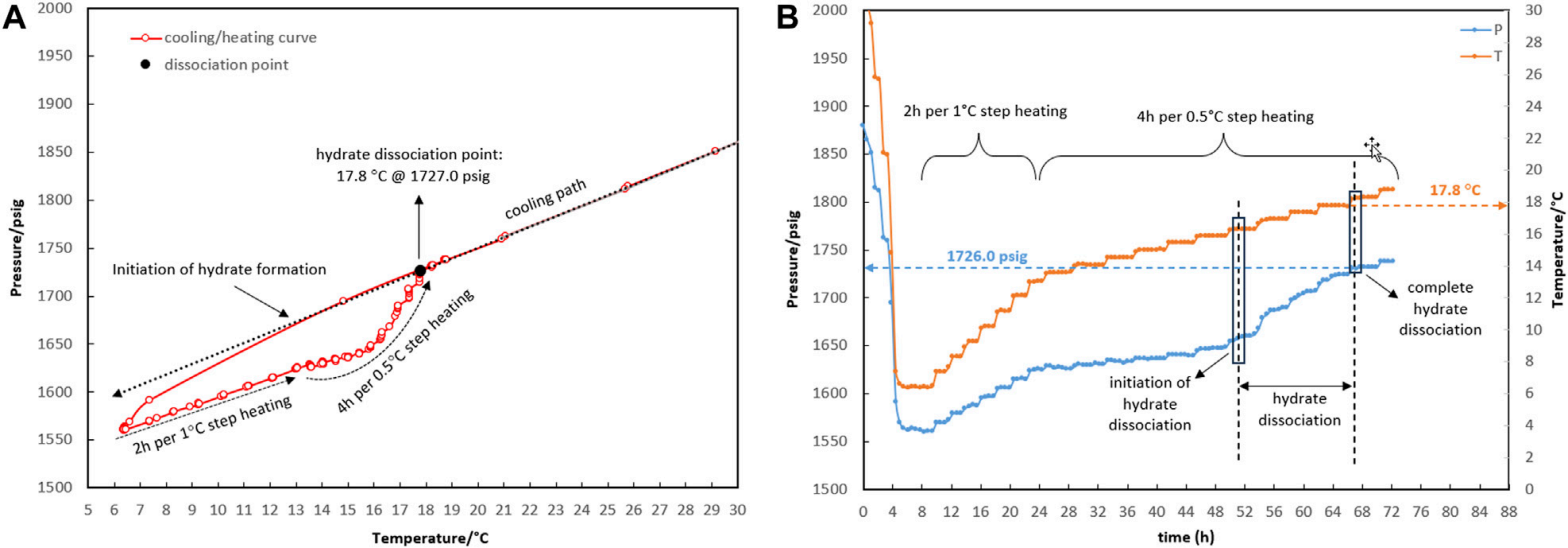
Four-phase hydrate equilibrium point (L_w_-L_HC_-H-G) determination with the water-to-hydrocarbon volume ratio of 1:6 in the presence of a 0.5% corrosion inhibitor and absence of kinetic hydrate inhibitor. **(A)** P-T diagram. **(B)** Pressure and temperature profiles.

**FIGURE 3:**
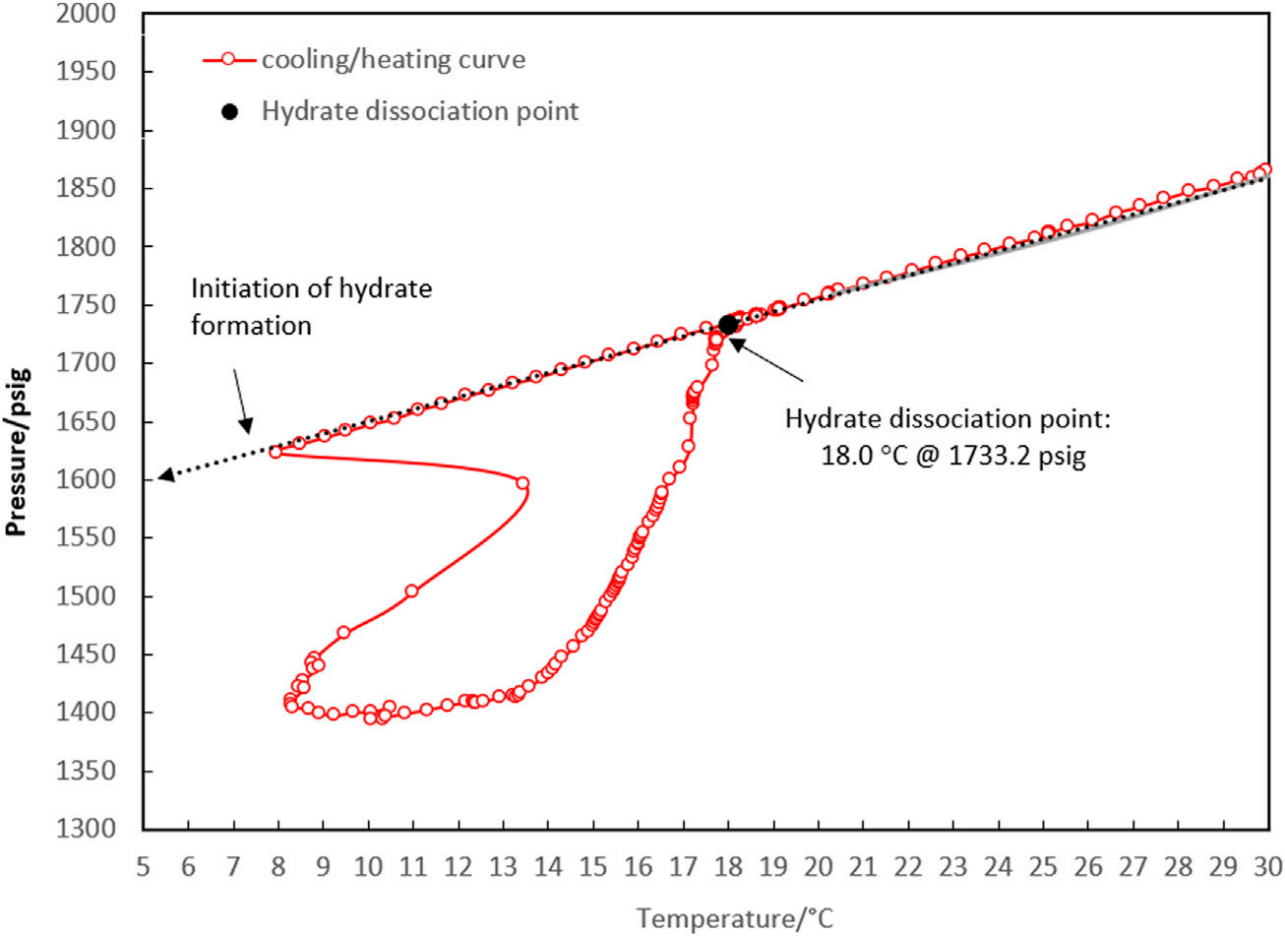
P-T diagram for determining the four-phase hydrate equilibrium point (L_w_-L_HC_-H-G) with a water-to-hydrocarbon volume ratio of 1:1 in the presence of a 0.5% corrosion inhibitor and absence of kinetic hydrate inhibitor.

Figures 2, 3 demonstrate the three/four-phase hydrate equilibrium point (L_w_-L_HC_-H-G) determined in the presence of a 0.5% corrosion inhibitor and the absence of a kinetic hydrate inhibitor for two systems with different water-to-hydrocarbon volume ratios of 1:6 and 1:1, respectively.

The initial temperature and pressure of the L_w_-L_HC_-G three-phase were considered to be 30.4°C and 1,864.9 psig. The system was allowed to form hydrate during the cooling rate of 5°C/h (temperature bath from 30°C to 5°C). The thermodynamic point was obtained from the equilibrium decomposition of hydrate during a two-stage heating path. During the first stage, up to 13.5°C, the system was given 2 hours to reach equilibrium pressure for every degree Celsius increase in temperature. However, according to Figure 2, the linear trend of the temperature and pressure profiles in the first stage indicates that the system was undergoing gas expansion rather than hydrate decomposition. Spending 4 h for every 0.5-degree Celsius increase in the second stage came next. Only gas expansion was observed up to a temperature of 16.3°C, after which a surge in pressure changes was indicated as hydrate dissociation initiation. The heavier components accumulate in the gas mixture as the hydrate crystals grow, changing its composition. This was essentially due to competition between the lighter and heavier components of the gas mixture for entry into the hydrate structure’s cages; the gas composition will not return to its initial value until all the hydrate has been dissociated. The hydrate dissociation continued until the temperature reached 17.8°C, at which the heating and cooling curves intersected as a result of the complete hydrate decomposition. The system’s temperature and pressure conditions were thus restored to the three-phase equilibrium of hydrocarbon, water, and gas. Thence, the pressure change is merely attributable to gas expansion as the system’s temperature rose. The equilibrium point was estimated at 17.8°C and 1,727 psig for the water-to-hydrocarbon volume ratio of 1:6.

According to the method described above, the equilibrium point of the 1:1 volume ratio of water to condensate was measured at 18.0°C and 1,733.2 psig, which corresponded to the initial point of 30.4°C and 1,865.5 psig.

Comparing the temperature and pressure curves in Figures 2A, 3, it can be observed that more hydrate accumulated with a water-to-hydrocarbon volume ratio of 1:1 rather than 1:6. Since the linear trend of pressure changes in terms of temperature (both during cooling and heating) demonstrated the expansion and compression of the gas phase, the slope of this linear section was relative to the moles of the gas phase. Figure 3 shows that the linear slope during cooling (moles of gas phase before hydrate formation) is much higher than during heating (moles of gas phase after hydrate formation) in the system, with a water-hydrocarbon volume ratio of 1:1. In contrast, Figure 2A shows the system with a water-to-hydrocarbon volume ratio of 1:6.

### 3.2 Impact of water-hydrate-memory degree on secondary hydrate formation

A set of six tests (R1–R6) were designed and executed to establish a procedure that benefitted from the presence of water-hydrate memory to improve the reproducibility of hydrate re-formation.

Water-hydrate memory is established in a system where hydrate is formed at least once and then decomposes. The main question is whether it is possible to identify a procedure for achieving reproducible water-hydrate memory applied to the next hydrate re-formation. It is expected that the largest degree of water-hydrate memory occurs around the hydrate equilibrium phase boundary when the last particle of hydrate has disintegrated. However, moving the system away from the hydrate equilibrium phase boundary through heating lowers the degree of water-hydrate memory and subsequently increases the induction time of hydrate reformation through secondary cooling.

At the start of each test, the system was typically maintained at three temperatures outside the hydrate region (30, 25, and 20°C) where only three phases of gas, hydrocarbon, and water are in equilibrium. These three operating points depict the linear temperature and pressure changes according to the Complete Gases, which leads to better detection of the onset of hydrate formation during the cooling path.

**FIGURE 4:**
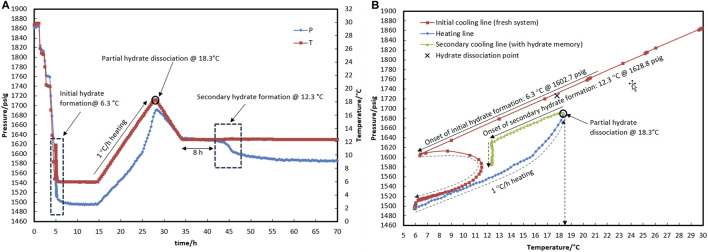
Hydrate formation/dissociation path of test R1 to create water memory with a water-to-hydrocarbon volume ratio of 1:6 in the presence of 0.5% corrosion inhibitor and 1.25% KHI; heating target temperature, 18.3°C. [**(A,B)** refer to P-t/T-t and P-T plots, respectively].

In test R1 (see temperature and pressure profiles in Figures 4A,B), a fresh system with no water-hydrate memory was cooled to 6.3°C (corresponding to a bath temperature of 5.0°C at a rate of 15°C/h), where hydrate formation was observed. Based on the equilibrium hydrate temperature of 17.8°C (Section 3.1), the target temperature of heating (with a rate of 1°C/h) was considered to be 18.3°C to ensure the highest degree of water-hydrate memory. Although it was observed that hydrate dissociation up to 18.3°C resulted in 1,727 psig for the control system with no hydrate inhibitor (Figure 2A), the pressure of the system in the presence of a hydrate inhibitor was enhanced up to 1,670 psig, representative of partial hydrate dissociation. This phenomenon seems to result from the heating time not being sufficient to achieve equilibrium hydrate dissociation in the presence of a kinetic hydrate inhibitor.

**FIGURE 5:**
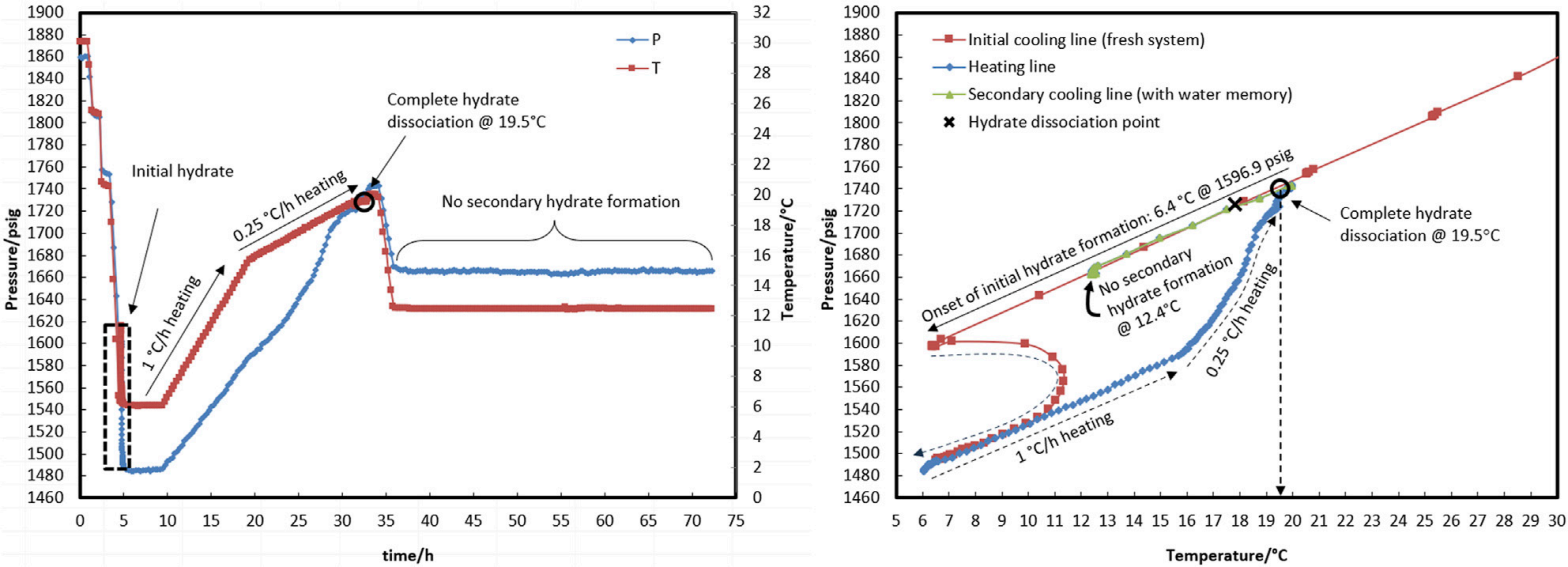
Hydrate formation/dissociation path of test R2 to create watery memory with the water-to-hydrocarbon volume ratio of 1:6 in the presence of a 0.5% corrosion inhibitor and 1.25% KHI; heating target temperature, 19.5°C.

To deal with this case in test R2 (see temperature and pressure profiles in Figures 5A,B), heating at a temperature higher than 15.5°C was continued not only at a rate of 1°C/h but also four times lower—a rate of 0.25°C/h—to allow enough time for hydrate decomposition. Interestingly, the complete dissociation of the hydrate did not occur at approximately 18.0°C, just as it did not in R1. At approximately 18.8°C in R2, the pressure reached 1,700 psig, and the rate of hydrate decomposition (according to pressure changes in Figure 5A) dropped significantly. It was shown that it was not practical to spend more time on achieving complete hydrate decomposition at a temperature of approximately 18.0°C. In turn, this happened at 19.5°C, approximately 1.8°C above the hydrate equilibrium temperature. The hydrate that forms in the presence of a kinetic hydrate inhibitor is more resistant to temperature changes during decomposition than the blank hydrate (Glénat et al., 2011; Ross et al., 2011). Higher temperatures are needed to completely decompose the hydrate. However, it was noted that the system’s temperature should be 2–4°C higher than the equilibrium temperature for the autoclave laboratory cells to preserve water-hydrate memory in the aqueous phase after complete dissociation of the hydrate (Duchateau et al., 2008).

Through the secondary cooling in test R1, hydrate formation was observed 8 h after the temperature reached 12.3°C due to pressure drop rather than any temperature peak (Figure 4A). This may have been associated with the elimination of the nucleation step and the direct development of hydrate crystal growth. However, no hydrate was found in R2 at 12.4°C for 35 h. At 19.5°C, the hydrate completely dissociated, and the temperature then rose to 20.5°C, which is 2.5°C above the equilibrium temperature, before the secondary cooling section.

**FIGURE 6:**
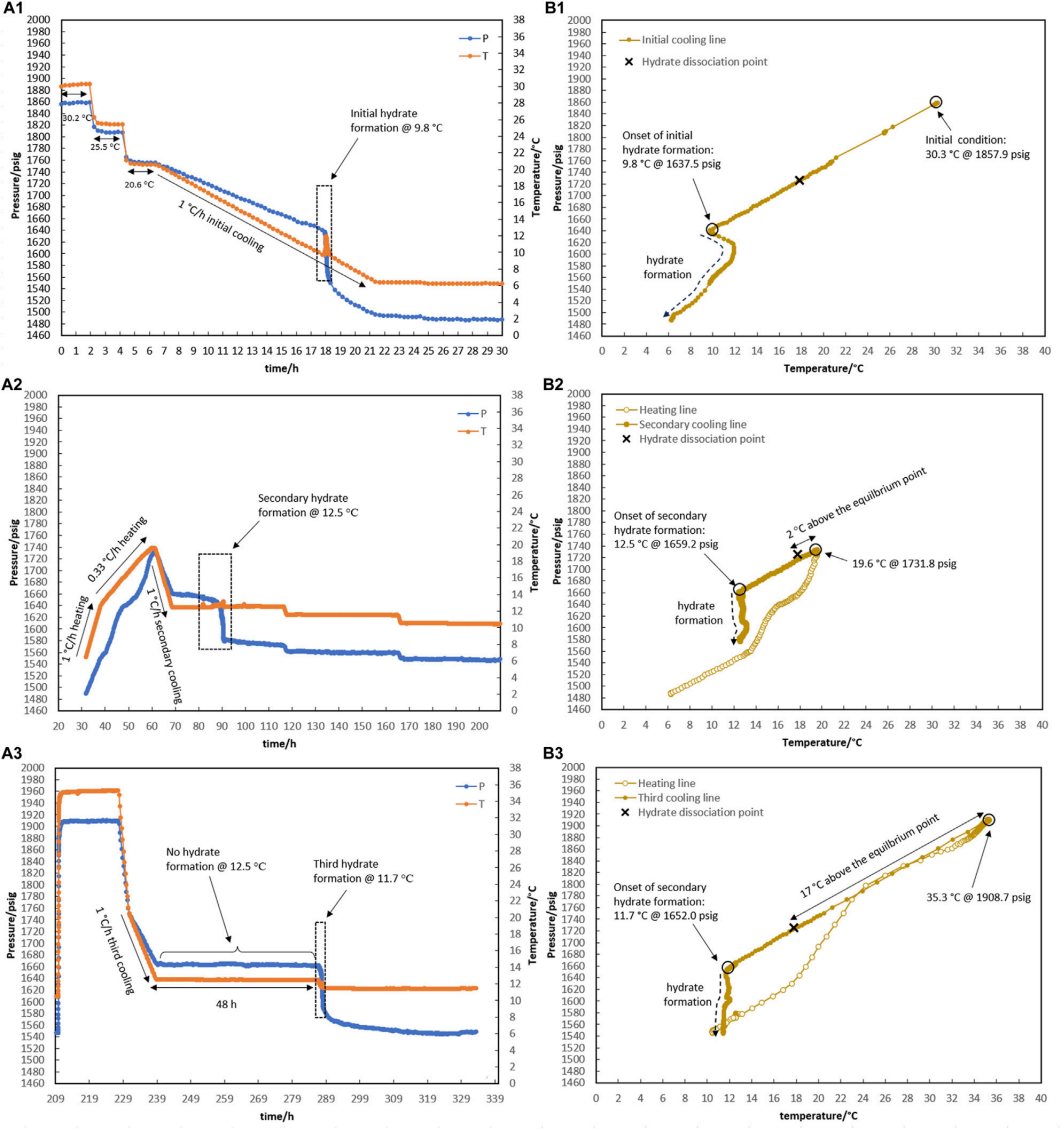
Hydrate formation/dissociation path of test R3 to create watery memory with the water-to-hydrocarbon volume ratio of 1:6 in the presence of 0.5% corrosion inhibitor and 1.25% KHI: heating target temperature: (1) fresh solution, (2) 19.6°C, and (3) 35.3°C. [**(A,B)** refer to P-t/T-t and P-T plots, respectively].

In test R3, the impact of water-hydrate memory created at 19.6°C (the lowest temperature at which the hydrate completely breaks apart when KHI is present) on the re-formation of the hydrate was studied. The temperature and pressure profiles are given in Figure 6.

For the initial cooling of 1°C/h of the fresh system in Figure 6A1, hydrate formation was postponed to 9.8°C. After that, hydrate gas consumption (i.e., a related pressure drops to hydrate growth) continued until the temperature inside the reactor reached 6.3°C within 4 h.

Like R2, the heating section was considered a two-stage process from 6.3°C to 12.6°C and from 12.6°C to 19.6°C, with a rate of 1°C and 0.33°C/h, respectively. Once more, complete hydrate decomposition at 19.6°C was obtained. After being at 19.6°C for 2 hours, secondary cooling started at a rate of 1°C/h, leading to 12.5°C, when the temperature remained constant. At an isothermal temperature of 12.5°C, the usual sign of hydrate formation (a pressure drop along with a temperature peak) appeared after 12.5 h (Figure 6A2). Although a major hydrate pressure drop occurred 6.5 h after the slow hydrate growth initiation, the hydrate’s temperature peak is incomparable in magnitude to what was observed in the fresh system.

It is important to mention that degrees of water-hydrate memory vary due to different target temperatures considered for complete hydrate dissociation through heating. The water-hydrate memory thus becomes weaker with a temperature increase during the dissociation of the hydrate. (Sefidroodi et al., 2013). An increase of 1°C in the target temperature for heating during hydrate decomposition resulted in a 35-h delay in hydrate formation in test R2 (target temperatures of 20.5°C in Figure 5A2), compared to R3 (target temperatures of 19.6°C in Figure 6A2), where hydrate developed after 12.5 h was at 12.5°C.

As shown in Figure 6A3, the heating target temperature was increased to 35°C during R3, and the system was maintained at this temperature for 20 h to reduce the degree of water-hydrate memory to its probable minimum value. Cooling occurred from 20°C to 12.5°C at a rate of 1°C/h, while no hydrate formation was observed in the system during the 48 h of being kept isothermal at 12.5°C. These indications highlight how different levels of water-hydrate memory may affect the risk of hydrate formation.

**FIGURE 7:**
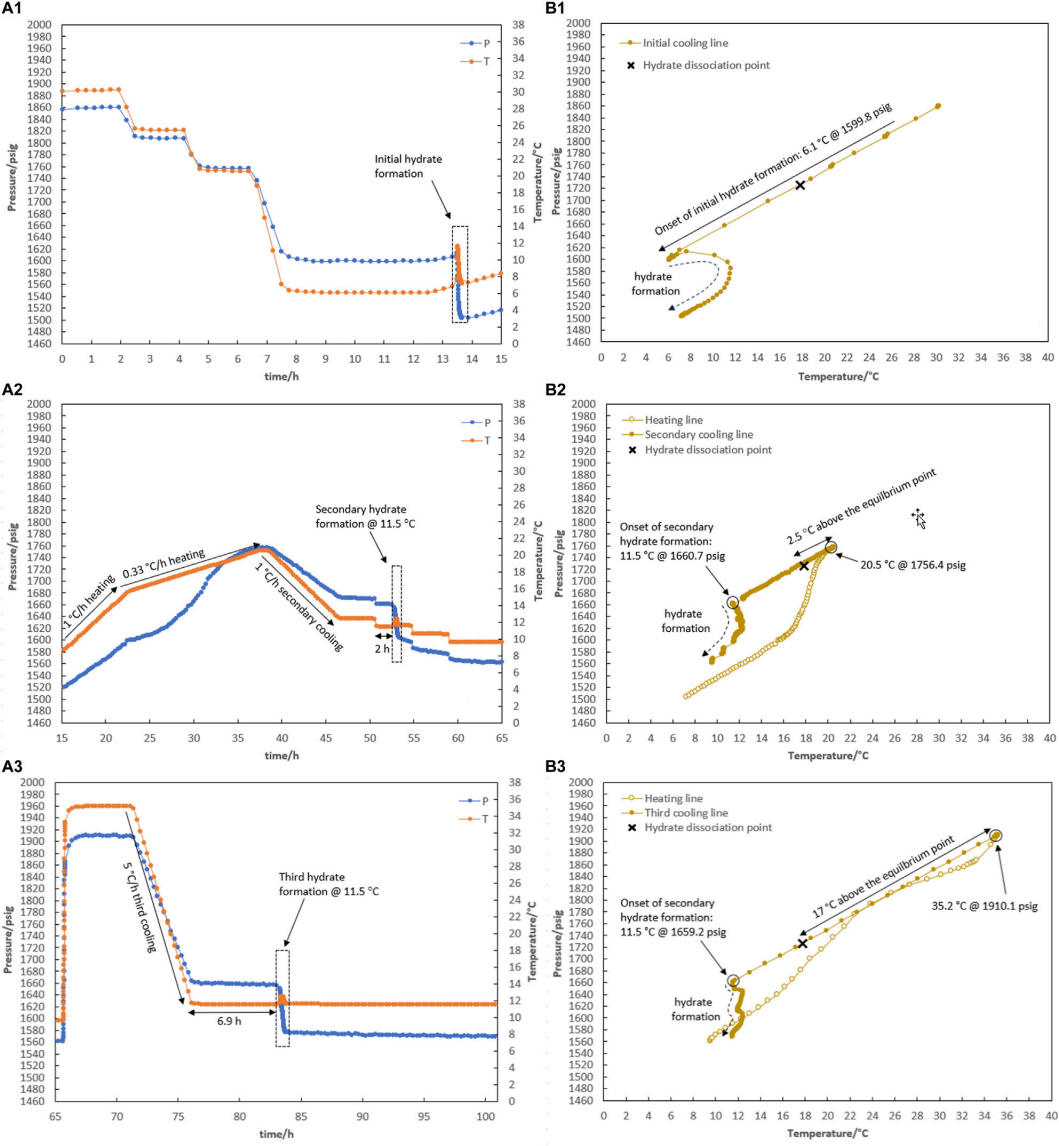
Hydrate formation/dissociation path of test R4 to create watery memory with the water-to-hydrocarbon volume ratio of 1:6 in the presence of 0.5% corrosion inhibitor and 1.25% KHI: heating target temperature: (1) fresh solution, (2) 20.5°C, and (3) 35.2°C. [**(A,B)** refer to P-t/T-t and P-T plots, respectively].

It is noteworthy that although hydrate did not form for 48 h at 12.5°C in R3, hydrate was formed immediately once the temperature decreased to 11.7°C. This raises the question: if a lower temperature is applied to the system instead of 12.5°C, how will the system respond to different degrees of water-hydrate memory against the risk of hydrate formation? Test R4 was designed and implemented in response to this question, and the results are given in Figure 7. Once hydrates were formed in a fresh solution at 6.1°C (Figure 7A1), they were dissociated using a two-step process that went from 6.3°C to 15.6°C and then from 15.6°C to 20.5°C at rates of 1°C and 0.33°C/h (Figure 7A2). Following secondary cooling at a rate of 1°C/h, 4-h step cooling occurred at 12.5, 11.5, 10.5, and 9.5°C. It was observed that hydrate formation started 2 h after being kept at 11.5°C (Figure 7A2). The system was then heated to 35°C and remained isothermal for approximately 5 h. By cooling 5°C/h, the system temperature decreased directly to 11.5°C. Hydrate formation was observed 6.9 h after the system reached 11.5°C (Figure 7A3).

Regardless of the degree of water-hydrate memory related to the heating target temperatures of 20.5°C and 35°C, it was observed that the performance of KHI overcame the risk of hydrate formation at 12.5°C. It was clear that water-hydrate memory had a bigger impact on the performance of KHI at a lower KHI evaluation temperature of 11.5°C than the induction times of 2 and 6.9 h for different levels of water-hydrate memory originating from heating target temperatures of 20.5°C and 35.0°C.

### 3.3 Impact of water-to-hydrocarbon volume ratio on the effectiveness of water-hydrate-memory degree

**FIGURE 8:**
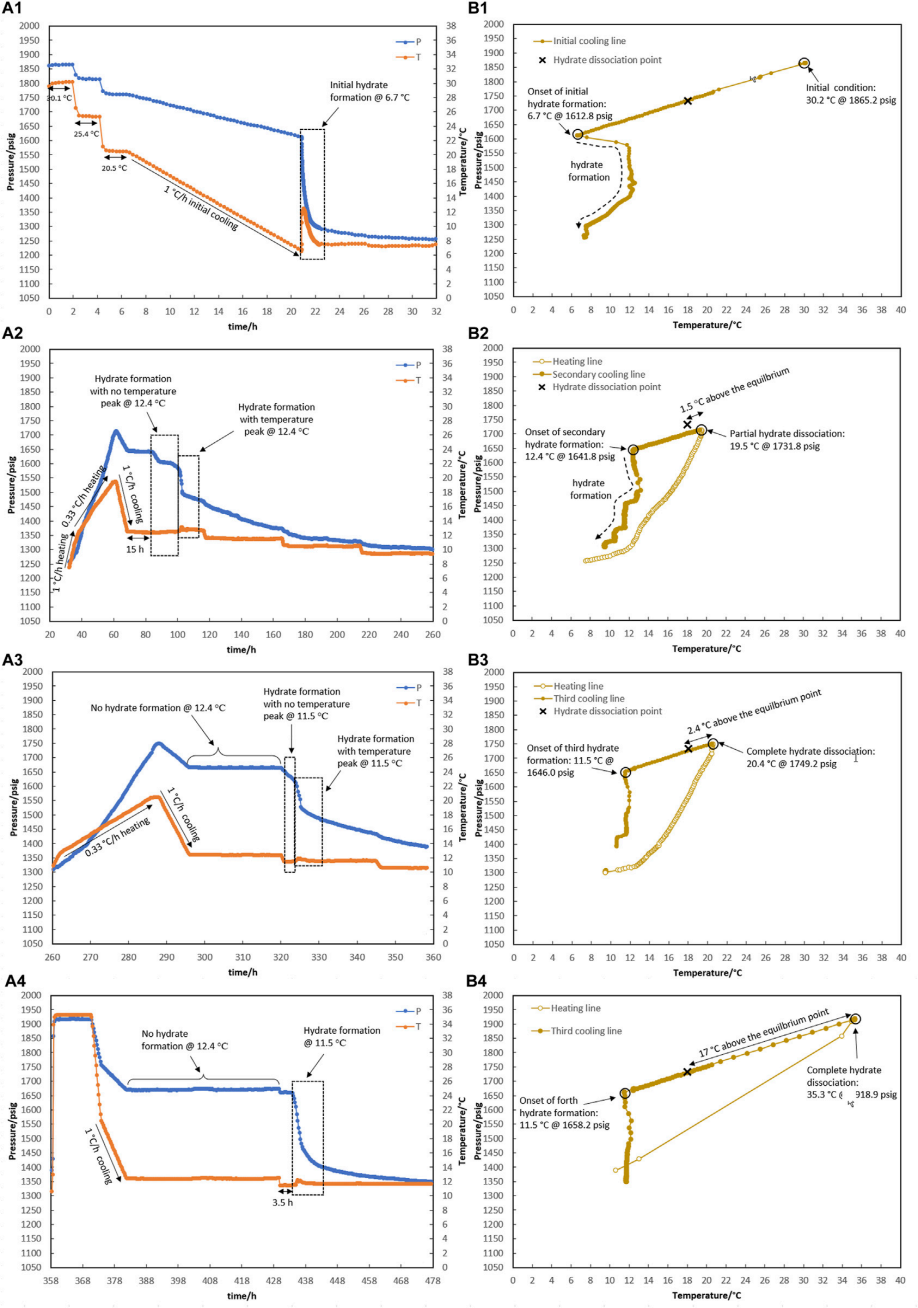
Hydrate formation/dissociation path of test R5 to create watery memory with the water-to-hydrocarbon volume ratio of 1:1 in the presence of 0.5% corrosion inhibitor and 1.25% KHI: heating target temperature: (1) fresh solution, (2) 19.6°C, (3) 20.4°C, and (4) 35.3°C. [**(A,B)** refer to P-t/T-t and P-T plots, respectively].

**FIGURE 9:**
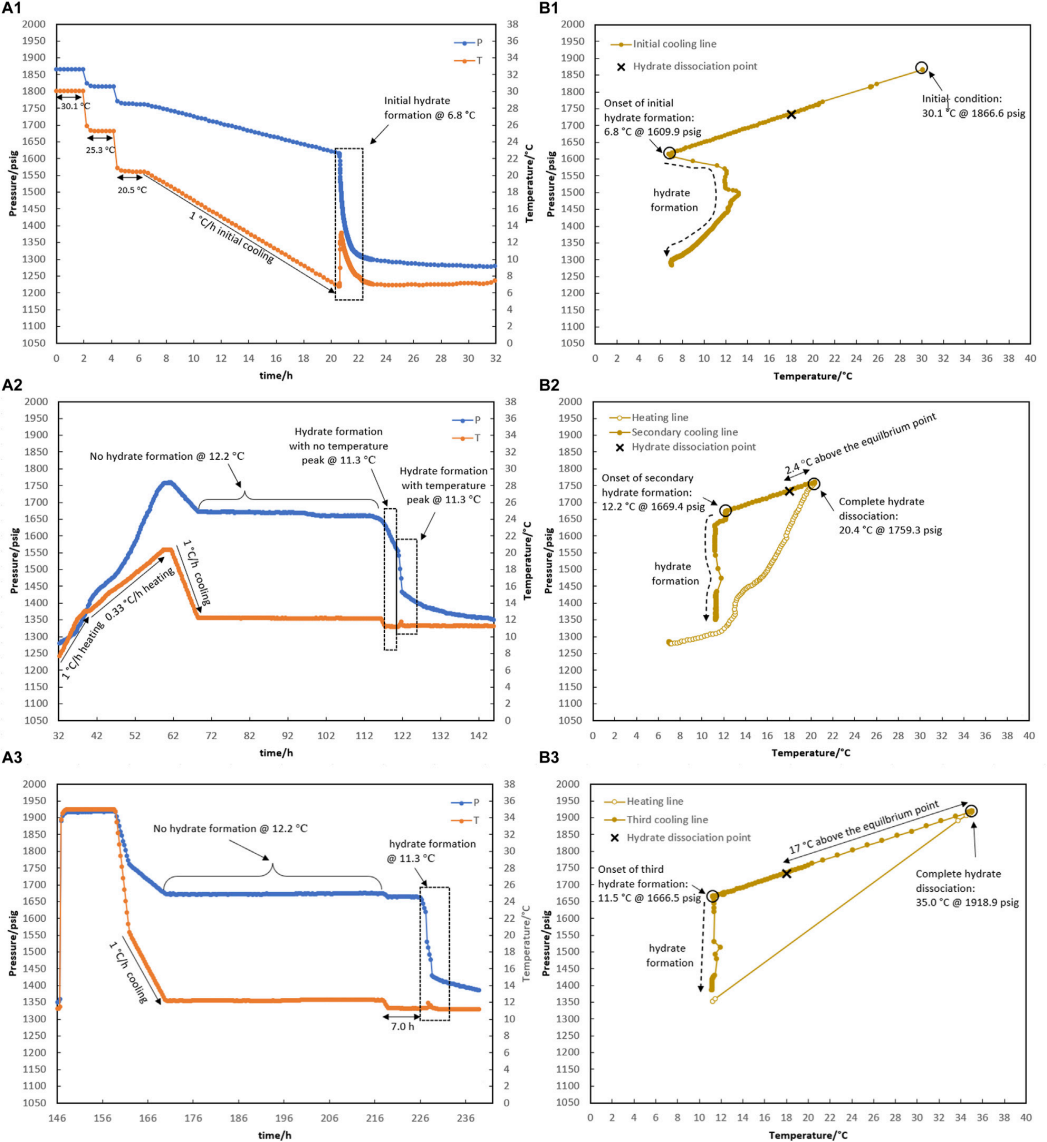
Hydrate formation/dissociation path of test R6 to create watery memory with the water-to-hydrocarbon volume ratio of 1:1 in the presence of 0.5% corrosion inhibitor and 1.25% KHI: heating target temperature: (1) fresh solution, (2) 20.4°C, and (3) 35.0°C. [**(A,B)** refer to P-t/T-t and P-T plots, respectively].

We investigated how the ratio of water to hydrocarbons affected the degree of water-hydrate memory and, later, hydrate re-formation. Two tests of R5 and R6 with a similar ratio of water to hydrocarbons of 1:1 were carried out Figures 8, 9.

As shown in Figure 8, the creation of water-hydrate memory based on the target temperatures of 19.5°C (1.5°C higher than the equilibrium temperature of 18.0°C measured in Section 3.1 for this system), 20.4°C, and 35.3°C were investigated in Test R5. First, it was observed that hydrate formed at 6.7°C while decreasing the temperature at a rate of 1°C/h through the initial cooling of the fresh system without any water-hydrate memory (Figure 8A1).

As mentioned in the prior section, hydrate decomposition was carried out in two consecutive stages at rates of 1°C and 0.33°C/h. Although the heating target temperature of 19.5°C was 1.5°C above the equilibrium temperature, the hydrate was not completely decomposed (Figure 8A2).

After secondary cooling from 19.5°C to 12.4°C, the first mild gas consumption without any temperature peak was observed after 15 h, keeping the system at 12.4°C. This took 14 h, and then a sudden pressure drop along with a temperature peak was observed, continuing for the next 48-h temperature steps of 11.5°C and 10.5°C (Figure 8A2). To eliminate the hydrate, the system was heated to 20.4°C, and the formation of hydrate was observed again during the secondary cooling. No hydrate was formed at 12.4°C for 24 h. As the temperature decreased to 11.5°C, hydrate formation was initiated immediately in two stages: a mild pressure drop without a temperature peak, followed by a sudden pressure drop with intense heat generation and simultaneous observation of the temperature peak (Figure 8A3).

Next, the water-hydrate memory was minimized by heating the system to 35°C and maintaining this temperature for 10 h. A third cooling at 1°C/h then led to a 48-h isothermal condition at 12.4°C (Figure 8A4). No hydrate formation was observed in the system at this temperature. In return, with the decrease in temperature to 11.5°C, hydrate growth was detected after 3.5 h. It should be noted that for this minimum water-hydrate memory, there was also a two-stage gas consumption mechanism in the presence of the inhibitor, although the duration of the mild pressure drop was very limited and, in this test, was less than half an hour.

As shown in Figure 9, test R6 was conducted to ensure that the outcomes of R5 for various water memories could be replicated. Identical to the onset of hydrate formation in the fresh system of test R5, hydrate formation also began at 6.8°C in R6 (Figure 9A1). In the case of water memories, heating to 20.4°C and 35.0°C and subsequent secondary cooling at a rate of 1°C/h led to an identical outcome as R5: first, the formation of hydrate was delayed at 12.2°C for 48 h, and then a two-stage growth mechanism of hydrate formation was also observed when the system cooled to 11.3°C (Figures 9A2,A3).

In this article, some representative points about the water-hydrate-memory effect were observed and can be highlighted. First, once the hydrate completely decomposed, the temperature and pressure of the system return to the equilibrium values for the water and gas phases (i.e., the overlapping of the cooling and heating curves). Although it seems that all the gas molecules that entered the hydrate structure fully transitioned back into the gas phase, the hydrate formation from this water was not the same as the hydrate formation from fresh water that had not participated in the hydrate structure.

According to Wei and Maeda (2023) who reviewed the mechanisms proposed so far for the water-hydrate-memory effect on hydrate nucleation, it seems that the “interfacial nanobubble” hypothesis is consistent with these observations. In addition, higher amounts of nanobubbles in a 1:1 water-to-hydrocarbon volume ratio compared to a 1:6 ratio control the development of crystal growth, being a two-stage gas consumption process regardless of the water-hydrate-memory degree. In Figures 8A.2, 8A.3, hydrate growth begins with a mild pressure decrease and no temperature rise, followed by a more severe pressure drop and a small temperature peak. However, the first stage was faster by lowering the water-hydrate-memory degree due to a decrease in nanobubble amounts at higher system temperatures (compare Figures 8A.2, 8A.3).

Furthermore, when the system cooled again after hydrate decomposition, a small if not negligible temperature increase was observed at the macroscopic onset of hydrate growth (see Figures 6B1,B2, for example), which is remarkably similar to the observations of hydrate growth when a small amount of hydrate remained before the system re-cooled (Figure 4B). We propose that the water released from hydrate decomposition has a less disordered molecular arrangement, similar to the molecular arrangement of water in equilibrium with the hydrate phase during hydrate growth, resulting in less energy release during hydrate crystal formation than hydrate formation from fresh water. As a result, the system’s temperature rises at a slower rate during the water-to-hydrate conversion.

It seems that the presence of nanobubbles (based on the “interfacial nanobubble” hypothesis) together with a more regular molecular arrangement of water phase can explain the water-hydrate memory effect on the nucleation time decrease observed in hydrate formation after re-cooling of this system compared to fresh water.

### 3.4 Improved induction time procedure for evaluating the kinetic hydrate inhibitor

Given the background information from the earlier sections on how to make water-hydrate memory in the lab, an investigation was conducted to assess the effectiveness of utilizing it in conjunction with the induction time (IT) technique to evaluate KHIs in order to make the results more consistent.

According to the composition of the gas phase in this work, a bath temperature program was designed and followed to evaluate the performance of KHI using an induction time-based technique in conjunction with the water-hydrate memory—the hydrate memory effect (HME) method, as below. However, steps 1 and 4 were just followed for applying the IT method (as an example).

1. Keep the system at the phase equilibrium of gas, water, and liquid hydrocarbons (if any) out of the hydrate zone.
  ○ 2 h at 30°C.
  ○ Lower temperature to 25°C at rate of 25°C/h (5°C lowering within 12 min).
  ○ 2 h at 25°C.
  ○ Lower temperature to 20°C at 25°C/h.
  ○ 2 h at 20°C.
2. Applying subcooling resulted in hydrate formation.
  ○ Reduce temperature to 5°C at 15°C/h.
  ○ 10 h at a 5°C.
3. Complete hydrate dissociation to create water-hydrate memory.
  ○ Temperature increase from 5°C to 15°C at rate of 1°C/h.
  ○ Temperature increase from 15°C to the water-hydrate-memory target temperature (a minimum of 2–3°C above hydrate equilibrium measured in the laboratory to ensure complete hydrate dissociation) at 0.33°C/h.
  ○ 2 h at the water-hydrate memory target temperature.
4. Seek hydrate formation in the presence of water-hydrate memory.
  ○ Secondary cooling from 20°C to the bath temperature ensures the KHI evaluation target temperature in the vessel, at 1°C/h.
  ○ 48 h at the KHI evaluation target temperature.

Table 2 shows the results of hydrate risk evaluation in the presence of the KHI using both IT and HME methods for the system with a water-to-hydrocarbon volume ratio of 1:6 and the presence of 0.5% corrosion inhibitor and 1.25% KHI. We observed that for the KHI evaluation target temperature range of 12.3–12.4°C, the variance of the results is significant, making it impossible to definitively conclude that this inhibitor can effectively postpone hydrate formation for 48 h. Further repetition is needed to establish a conclusive result. The repeatability of the results through the HME method is confirmed for three replications, regardless of the degree of water-hydrate memory.

**TABLE 2:** Results of KHI performance evaluation with IT (induction technique) and HME (hydrate memory effect) methods for the system with the water-to-hydrocarbon volume ratio of 1:6 in the presence of 0.5% corrosion inhibitor and 1.25% KHI.

Runs	Method	Water-hydrate-memory target temperature (°C)	KHI evaluation target temperature (°C)	Hydrate formation	Induction time (h)	Mean induction time (h)	Relative standard deviation percentage (%)
1	IT	-	12.3–12.4	✕	>48	*	-
				✓	33		
				✕	>48		
2	HME	20.3–20.4		✕	>48	>48	-
				✕	>48		
				✕	>48		
3		35.0–35.3		✕	>48	>48	-
				✕	>48		
				✕	>48		
4	IT	-	11.5–11.7	✓	0.0	0.97 ± 0.90	94
				✓	1.1		
				✓	1.8		
5	HME	20.3–20.4		✓	0.2	0.13 ± 0.06	43
				✓	0.1		
				✓	0.1		
6		35.0–35.3		✓	6.1	5.47 ± 0.57	10
				✓	5.3		
				✓	5.0		

* Needs replication.

Moreover, hydrate formation was achieved by carrying out both the IT and HME methods while lowering the target temperature to 11.5–11.7°C. Based on a comparison of the relative standard deviation percent, it is clear that applying water-hydrate-memory yields more repeatable data on isothermal induction time.

## 4 Conclusion

There is a trade-off between accurate results and time and money expended when comparing two common and well-known methods—the induction time-based technique and the hydrate crystal growth method—which are used in the literature to test gas hydrate kinetic inhibitors in the lab. Based on this, we aimed to introduce a fast method that was still accurate enough to determine hydrate inhibition in the lab to be acceptable to industry. Of course, since the nature of the multiphase flow in the lines increases the probability of hydrate nuclei formation and decomposition, this method seems to be closer to simulating the fluid’s behavior in real conditions.

We thus examined the impact of water-hydrate memory on improving the accuracy and repeatability of the results of the simple induction-time-based technique. Water-hydrate memory was created as a result of the complete decomposition of hydrate in the system. Since accurate knowledge of the hydrate equilibrium point is required for this purpose, the phase hydrate equilibrium point (L_w_-L_HC_-H-G) was determined at 17.8°C and 18.0°C in the presence of a 0.5% corrosion inhibitor and the absence of a kinetic hydrate inhibitor for two systems with different water-to-hydrocarbon volume ratios of 1:6 and 1:1, respectively.

Tests R1, R2, R3, and R4 investigated how well the factors that increase or decrease water-hydrate memory evaluate the performance of KHI. These factors include the heating rate and the target temperature of heating. The researchers concluded that full hydrate dissociation could only occur at temperatures higher than the hydrate equilibrium temperature. This is because a kinetic hydrate inhibitor made hydrate dissociation more difficult than the blank system. In this work, complete hydrate dissociation occurred at 19.5°C in the presence of KHI, considering 17.8°C as the hydrate equilibrium temperature for a system with water-to-hydrocarbon volume ratios of 1:6; the strongest water-hydrate memory was observed at this temperature. This was expressed in light of the observation that, as a result of water-hydrate memory maintained at 19.6°C, hydrate formed isothermally after 12.5 h at the KHI evaluation temperature of 12.5°C. On the other hand, hydrate formation was postponed for more than 30 h under the effect of water-hydrate memory maintained at 20.5°C. However, similar results on delaying hydrate formation were observed for water-hydrate memory maintained at temperatures up to 35.0°C. Moreover, it was clear that water-hydrate memory had a bigger impact on the performance of KHI at a lower KHI evaluation temperature of 11.5°C, comparing induction times of 2 and 6.9 h for different levels of water-hydrate memory that originate from heating target temperatures of 20.5°C and 35.0°C. Moreover, it was found that hydrates formed at a lower KHI evaluation target temperature of 11.5°C, regardless how much water-hydrate memory there was. Comparing the induction times for 2°C and 6.9°C measured, respectively, for heating target temperatures of 20.5°C and 35.0°C, this emphasizes that the system is less likely to allow hydrates to form when there is less water-hydrate memory.

As a result of investigating the effect of the water-to-hydrocarbon ratio (1:6 vs 1:1), a two-stage gas consumption mechanism was discovered by using water-hydrate memory in the presence of kinetic hydrate inhibitors, with a mild pressure drop without a temperature peak followed by a sudden pressure drop with intense heat generation and simultaneous observation of a temperature peak. Lowering the strength of water-hydrate memory at a higher heating target temperature (20.5°C vs 35.0°C) limited the stage of mild pressure drop to even less than half an hour, which can be neglected compared to the second stage of related hydrate growth.

Finally, a procedure was developed based on the background information from the earlier sections to compare the consistency of the results, which originates from the conjunction of water-hydrate memory with the induction time technique. Using three replications at the KHI evaluation target temperature of 12.3–12.4°C, we concluded that the HME method (an induction time-based technique in conjunction with water-hydrate memory) resulted in a more conclusive decision on evaluating KHI than the IT method (an induction-time-based technique). On the other hand, a comparison of the relative standard deviation percentage for isothermal induction time measured at the KHI evaluation target temperature of 11.5°C–11.7°C revealed that more repeatable data could be obtained by applying water-hydrate memory.

Although this research resulted from an industrial study using industrial samples, and although we were aware that different sampling temperatures and pressures of the field than used in this research can result in variable gas composition through laboratory testing, the noteworthy results obtained have encouraged us to develop this research for a synthetic gas with a constant composition (no conversion to liquid hydrocarbon phase during the testing condition) in the coming year.

## Data Availability

The datasets presented in this article are not readily available because the dataset must be accessed in coordination with the authors. Requests to access the datasets should be directed to MM-T, m.mohammadtaheri@yahoo.com.
